# Real-Time Full-Color Overlay Indocyanine Green Navigation for Laparoscopic Deroofing of Liver Cysts

**DOI:** 10.70352/scrj.cr.25-0711

**Published:** 2026-03-06

**Authors:** Koya Yoshida, Satoru Seo, Masaki Aida, Yasuhiro Kawanishi, Kazune Fujisawa, Masaya Munekage, Hiromichi Maeda, Hiroyuki Kitagawa, Tsutomu Namikawa

**Affiliations:** Department of Surgery, Kochi Medical School, Kochi University, Nankoku, Kochi, Japan

**Keywords:** indocyanine green fluorescence imaging, laparoscopic deroofing, optimal resection line, real-time navigation

## Abstract

**INTRODUCTION:**

Laparoscopic deroofing is the standard treatment for symptomatic liver cysts. Indocyanine green (ICG) fluorescence imaging has been effectively used to delineate the boundary between cysts and liver parenchyma; however, intermittent switching between fluorescence and normal light modes is time-consuming and requires memorizing the cutting lines along a curved surface, which can be burdensome during surgery.

**CASE PRESENTATION:**

We report two cases of laparoscopic deroofing of liver cysts using the PINPOINT and 1788 systems, which enable full-color visualization of ICG signals and real-time surgical navigation. The full-color overlay mode clearly defines the cyst–liver boundary, allowing surgeons to determine optimal resection lines and perform procedures simultaneously. This enhances surgical precision, reduces intraoperative stress, and may improve outcomes. Both patients had uneventful recoveries and were discharged on POD 8.

**CONCLUSIONS:**

Laparoscopic deroofing with real-time ICG navigation is a safe and accurate surgical technique, performed in a minimally invasive setting that facilitates efficient and precise liver cyst resection.

## Abbreviation

ICG
: indocyanine green

## INTRODUCTION

Liver cysts are a common benign liver lesion, occurring in approximately 2.5%–18% of individuals. It is more prevalent among women and older adults and causes symptoms in a minority of cases.^1,2)^ Symptoms such as abdominal pain, nausea, and vomiting frequently result from the mass effect of the cyst, often necessitating intervention. Currently, laparoscopic deroofing is widely adopted owing to superior short-term outcomes and comparable long-term recurrence rates, compared with open surgery.^3,4)^

Surgeons often emphasize the importance of balancing the following two outcome measures in deroofing: a small fenestration increases recurrence risk, whereas an excessively large fenestration increases the likelihood of intraoperative complications, including hemorrhage and bile duct injury. ICG fluorescence imaging has been advocated to delineate bile ducts for overcoming this issue,^5–7)^ since ICG is excreted in bile. Furthermore, it effectively distinguishes liver parenchyma from cyst walls,^8–10)^ enabling accurate evaluation of the resection line in fluorescence mode and safe fenestration in normal light mode.

However, surgeons truly require real-time navigation surgery that continuously identifies a safe resection line, reducing the need for frequent mode switching. This surgery is particularly valuable when the resection line follows a curved surface, as in liver cyst deroofing. Here, we report two cases of symptomatic liver cysts treated with real-time ICG navigation.

## CASE PRESENTATION

**Fig. 1:**
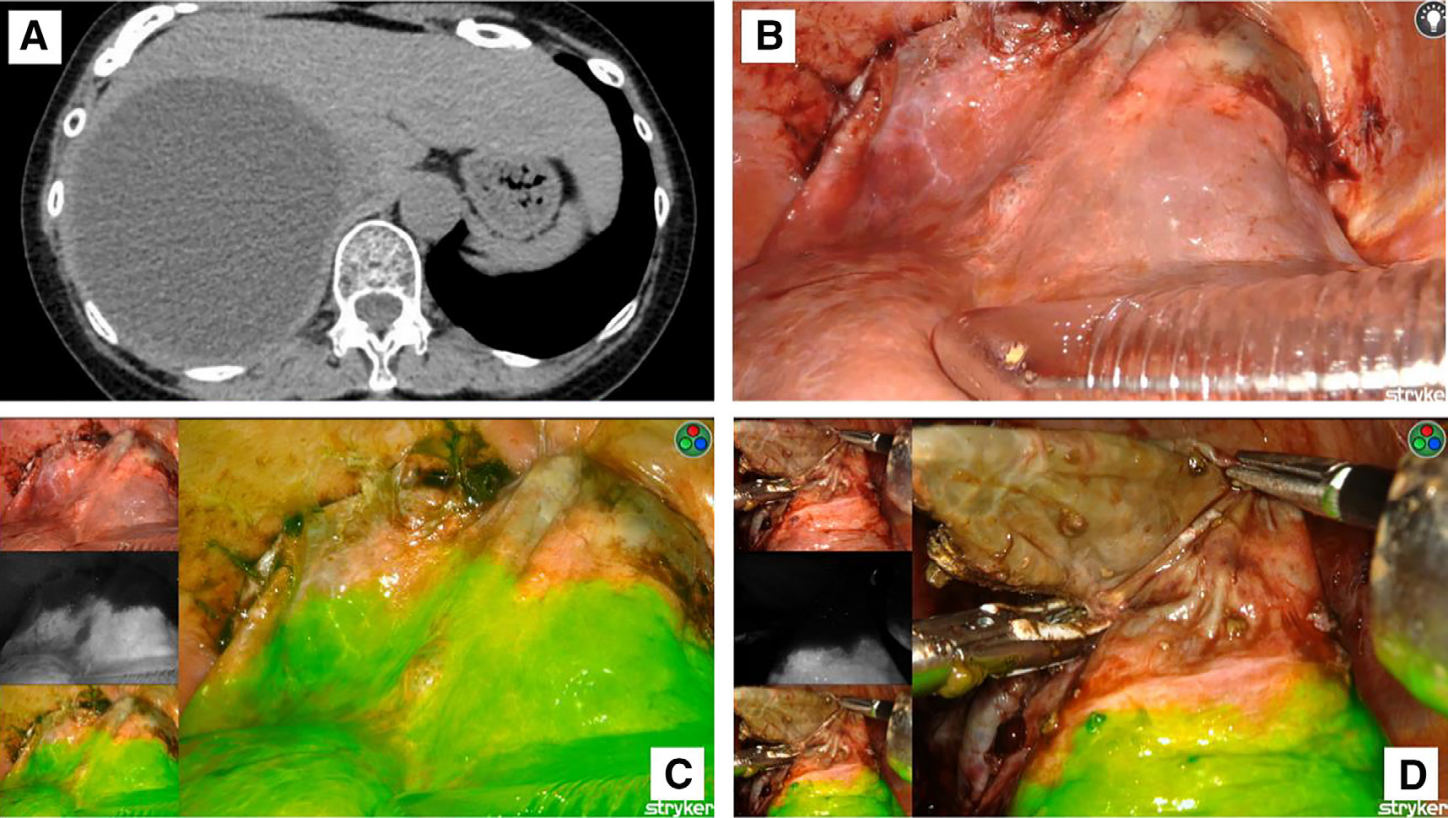
Preoperative CT and intraoperative findings of case 1. (**A**) Abdominal CT shows a large cystic lesion in the right hepatic lobe. The area where liver parenchyma covers the cyst is identifiable. (**B**) Under normal light observation, the boundary between the parenchyma and the cyst wall is unclear. The surgeon may choose to initiate the incision near the concave plane (above the tip of the port). (**C**) Full-color overlay mode indicates that the optimal deroofing line lies ventral to the concave plane (ICG-positive liver parenchyma is shown in green). (**D**) Surgery is performed safely under full-color overlay mode guidance. ICG, indocyanine green

**Fig. 2:**
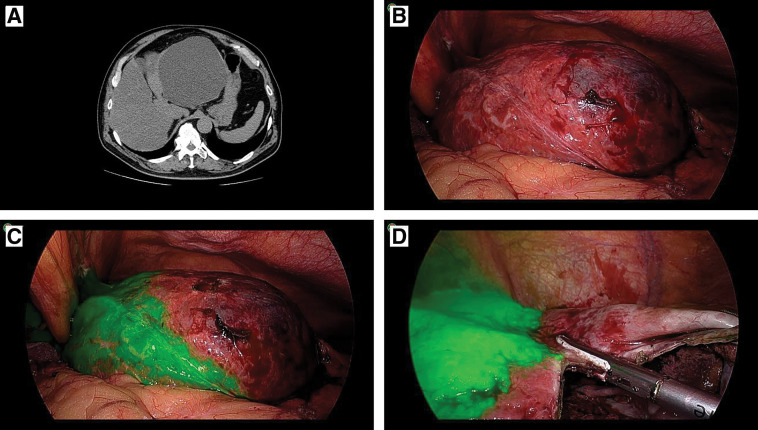
Preoperative CT and intraoperative findings of case 2. (**A**) Abdominal CT reveals a cystic lesion in the lateral segment of the liver, with a thin layer of parenchyma covering the cyst. (**B**) The boundary between the parenchyma and the cyst wall is not clearly visible under normal light. (**C**) Full-color overlay mode clearly delineates the appropriate line for deroofing (ICG-positive liver parenchyma is shown in green). (**D**) Surgery is conducted safely using the full-color overlay mode. ICG, indocyanine green

The first patient was a 72-year-old woman who presented with an 11-cm infectious liver cyst in the right hepatic lobe (**Fig. 1**). The second patient was a 64-year-old man with a 14-cm infectious liver cyst in the left hepatic lobe (**Fig. 2**). No evidence of malignancy was observed, including intracystic nodules on CT or MRI, or elevated tumor marker levels.

For the first patient, the PINPOINT Endoscopic Fluorescence Imaging System (Stryker, Kalamazoo, MI, USA) was used. Under general anesthesia and in the left lateral decubitus position, a camera port was placed at the umbilicus, with three other instrument ports positioned at the right costal margin, right lateral abdomen, and epigastrium. For the second patient, the 1788 system (Stryker) was used. In the supine position, a camera port was placed at the umbilicus with four additional ports placed in the left abdomen, left upper quadrant, right abdomen, and epigastrium. In both cases, LigaSure (Medtronic, Minneapolis, MN, USA) was used for cyst wall resection, and ICG (1.25 mg/body) was injected intravenously.

The delineation of the resection line relies on contrasting liver parenchyma, which has abundant blood flow, with the thin cyst wall, which has low blood flow. Thus, ICG was injected immediately before demonstrating the resection line because the administered ICG swiftly reaches the live. The full-color overlay mode clearly displayed the boundary between the cyst wall and liver parenchyma. Intraoperative ultrasonography was used to identify the area with the thinnest wall to ensure a safe incision site. After aspirating the cyst contents via puncture, the cyst wall was incised. Under normal light, the boundary between the cyst and parenchyma was unclear (**Figs. 1B** and **2B**). However, in full-color overlay mode, optimal resection lines were readily visible (**Figs. 1C** and **2C**). Resection was performed with a margin from the ICG-demarcated boundary to prevent bile duct injury or unexpected hemorrhage (**Figs. 1D** and **2D**).

After completion of deroofing (approximately 60 minutes after ICG injection, which was sufficient time for ICG to be excreted into the bile), we checked for bile leakage using ICG imaging.^11)^ The absence of bile leakage was confirmed by applying gauze to the cut surface of the cyst wall and observing the lack of ICG fluorescence on gauze.

Drainage catheters were placed via the epigastric port into the cyst cavities. The operation lasted 223 min for the first patient and 261 min for the second, with minimal blood loss in both cases. Both patients recovered without complications and were discharged on POD 8.

## DISCUSSION

Asymptomatic cysts typically require no intervention, while initial treatment for symptomatic cysts may include intravenous antibiotic therapy and ultrasound-guided percutaneous drainage with sclerotherapy.^12)^ However, sclerotherapy and aspiration are associated with high recurrence rates, often necessitating repeated procedures.^13)^ Surgical options for hepatic cysts include hepatic resection, cyst excision, deroofing (fenestration), and liver transplantation for polycystic liver disease.^14)^ The choice of intervention should be based on cyst size, location, infection status, and patient-specific factors. Since its introduction in 1991,^15)^ laparoscopic deroofing has become a preferred treatment for liver cysts because of its minimally invasive nature and favorable short- and long-term outcomes.^3,4)^

Laparoscopic deroofing, although effective, carries risks such as hemorrhage, bile duct injury, and bile leakage. When bile duct injury occurs intraoperatively, suturing may be required; however, the limited working space makes the procedure technically challenging and increases the risk of postoperative bile duct stricture. If bile leakage is identified postoperatively, endoscopic nasobiliary drainage may be necessary, often prolonging hospitalization. Intraoperative cholangiography is one method used to detect bile duct injuries; nevertheless, the procedure is time-consuming and requires direct ductal cannulation. ICG fluorescence imaging, benefiting from biliary excretion of ICG, offers a less invasive alternative to delineate bile ducts and detect bile leakage.^5–7)^ It also assists in identifying appropriate cutting lines during liver cyst deroofing.^8–10)^ Traditionally, ICG imaging has been used intermittently, offering limited support for continuous image-guided surgery.

Advancements in laparoscopic systems have made it possible to visualize ICG fluorescence in real time using a full-color overlay, eliminating the need for mode switching. The effectiveness of this approach has been documented in laparoscopic hepatectomy,^16)^ and we demonstrate its utility for liver cyst deroofing. Intraoperatively, weak or absent ICG fluorescence indicated cyst walls, whereas strong signals marked liver parenchyma. This contrast allowed for accurate determination of resection lines, which could be followed in real time without changing imaging modes. This technique is particularly useful when the resection surface is irregular or curved, as in liver cysts. By offering continuous visualization of the anatomic boundary, it reduces cognitive load and improves precision during resection. Additionally, maintaining the same imaging mode throughout the procedure simplifies workflow and enhances surgical safety. Limitations include the need for an ICG fluorescence imaging system, which may entail additional equipment costs, and the limited availability of such systems across institutions. However, in centers where ICG navigation is routinely used for laparoscopic hepatectomy,^16)^ the additional implementation cost is minimal. Moreover, as ICG fluorescence imaging is feasible on robotic platforms, this technique may be broadly applicable to robot-assisted and other hepatobiliary procedures.

## CONCLUSIONS

Real-time navigation using ICG fluorescence imaging facilitates safe, efficient, and precise laparoscopic deroofing of liver cysts. This technique minimizes the need for mode switching, reduces intraoperative stress, and may help prevent complications such as bile duct injury and hemorrhage. As demonstrated in our two cases, this method is a promising advancement in minimally invasive liver surgery.

## References

[1] Lantinga MA, Gevers TJ, Drenth JP. Evaluation of hepatic cystic lesions. World J Gastroenterol 2013; 19: 3543–54. 23801855 10.3748/wjg.v19.i23.3543 PMC3691048

[2] Kelly K, Weber SM. Cystic diseases of the liver and bile ducts. J Gastrointest Surg 2014; 18: 627–34; quiz 634. 24356979 10.1007/s11605-013-2426-8

[3] Qiu JG, Wu H, Jiang H, et al. Laparoscopic fenestration vs open fenestration in patients with congenital hepatic cysts: a meta-analysis. World J Gastroenterol 2011; 17: 3359–65. 21876626 10.3748/wjg.v17.i28.3359 PMC3160542

[4] Zhang JY, Liu Y, Liu HY, et al. Comparison of the recurrence rates of nonparasitic hepatic cysts treated with laparoscopy or with open fenestration: a meta-analysis. Surg Laparosc Endosc Percutan Tech 2018; 28: 67–72. 29528948 10.1097/SLE.0000000000000516

[5] Tanioka N, Maeda H, Shimizu S, et al. Indocyanine green fluorescence-guided laparoscopic deroofing of a liver cyst: a case report. Asian J Endosc Surg 2022; 15: 359–62. 34643051 10.1111/ases.12999

[6] Hanaki T, Yagyu T, Uchinaka E, et al. Avoidance of bile duct injury during laparoscopic liver cyst fenestration using indocyanine green: a case report. Clin Case Rep 2020; 8: 1419–24. 32884766 10.1002/ccr3.2840 PMC7455442

[7] Tanaka M, Inoue Y, Mise Y, et al. Laparoscopic deroofing for polycystic liver disease using laparoscopic fusion indocyanine green fluorescence imaging. Surg Endosc 2016; 30: 2620–3. 26416378 10.1007/s00464-015-4526-x

[8] Une N, Fujio A, Mitsugashira H, et al. Laparoscopic liver cyst fenestration with real-time indocyanine green fluorescence-guided surgery: a case report. J Surg Case Rep 2021; 2021: rjab196. 34025978 10.1093/jscr/rjab196 PMC8128400

[9] Shimagaki T, Itoh S, Toshida K, et al. Prevention of bile duct injury using indocyanine green fluorescence in laparoscopic liver cyst fenestration for giant liver cyst: a case report. J Surg Case Rep 2022; 2022: rjac479. 36285169 10.1093/jscr/rjac479 PMC9581503

[10] Nakamura G, Asai K, Watanabe R, et al. Two cases of laparoscopic deroofing of giant liver cysts using indocyanine green fluorescence imaging. Asian J Endosc Surg 2024; 17: e13308. 38622489 10.1111/ases.13308

[11] Shibata H, Aoki T, Koizumi T, et al. The efficacy of intraoperative fluorescent imaging using indocyanine green for cholangiography during cholecystectomy and hepatectomy. Clin Exp Gastroenterol 2021; 14: 145–54. 33958888 10.2147/CEG.S275985 PMC8096340

[12] Yoshimoto T, Takajo T, Iijima H, et al. Comparison of endoscopic ultrasound-guided drainage and percutaneous drainage combined with minocycline sclerotherapy for symptomatic hepatic cysts: a retrospective study. Medicine (Baltimore) 2024; 103: e37677. 38552057 10.1097/MD.0000000000037677 PMC10977566

[13] Hartmann KD, Mortensen FV. Surgical treatment of symptomatic simple liver cysts. Dan Med J 2022; 70: A08220469. 36629296

[14] Gomez A, Wisneski AD, Luu HY, et al. Contemporary management of hepatic cyst disease: techniques and outcomes at a tertiary hepatobiliary center. J Gastrointest Surg 2021; 25: 77–84. 33083858 10.1007/s11605-020-04821-1 PMC7850990

[15] Fabiani P, Katkhouda N, Iovine L, et al. Laparoscopic fenestration of biliary cysts. Surg Laparosc Endosc 1991; 1: 162–5. 1669396

[16] Seo S, Ogiso S, Toda R, et al. Intraoperative indocyanine green imaging facilitates optimal surgical margin for colorectal liver metastasis with preoperatively undetected intrabiliary tumor growth. J Hepatobiliary Pancreat Sci 2022; 29: e48–9. 34403558 10.1002/jhbp.1037

